# Geographic Variation Did Not Affect the Predictive Power of Salivary Microbiota for Caries in Children With Mixed Dentition

**DOI:** 10.3389/fcimb.2021.680288

**Published:** 2021-06-18

**Authors:** Shanshan Li, Shi Huang, Yi Guo, Ying Zhang, Lijuan Zhang, Fan Li, Kaixuan Tan, Jie Lu, Zhenggang Chen, Qingyuan Guo, Yongping Tang, Fei Teng, Fang Yang

**Affiliations:** ^1^ School of Stomatology, Qingdao University, Qingdao, China; ^2^ Department of Pediatrics and Center for Microbiome Innovation at Jacobs School of Engineering, University of California, San Diego, CA, United States; ^3^ Department of Computer Science and Technology, The Key Laboratory of Embedded System and Service Computing, Ministry of Education, Tongji University, Shanghai, China; ^4^ Stomatology Department, Women & Children’s Health Care Hospital of Linyi, Linyi, China; ^5^ Stomatology Center, Qingdao Municipal Hospital, Qingdao, China; ^6^ Single-Cell Center, Qingdao Institute of Bioenergy and Bioprocess Technology, Chinese Academy of Sciences, Qingdao, China

**Keywords:** caries, geography, saliva microbiota, mixed dentition, diagnosis models

## Abstract

Dental caries is one of the most prevalent chronic oral diseases, affecting approximately half of children worldwide. The microbial composition of dental caries may depend on age, oral health, diet, and geography, yet the effect of geography on these microbiomes is largely underexplored. Here, we profiled and compared saliva microbiota from 130 individuals aged 6 to 8 years old, representing both healthy children (H group) and children with caries-affected (C group) from two geographical regions of China: a northern city (Qingdao group) and a southern city (Guangzhou group). First, the saliva microbiota exhibited profound differences in diversity and composition between the C and H groups. The caries microbiota featured a lower alpha diversity and more variable community structure than the healthy microbiota. Furthermore, the relative abundance of several genera (e.g., *Lactobacillus, Gemella*, *Cryptobacterium* and *Mitsuokella*) was significantly higher in the C group than in the H group (*p*<0.05). Next, geography dominated over disease status in shaping salivary microbiota, and a wide array of salivary bacteria was highly predictive of the individuals’ city of origin. Finally, we built a universal diagnostic model based on 14 bacterial species, which can diagnose caries with 87% (AUC=86.00%) and 85% (AUC=91.02%) accuracy within each city and 83% accuracy across cities (AUC=92.17%). Although the detection rate of *Streptococcus mutans* in populations is not very high, it could be regarded as a single biomarker to diagnose caries with decent accuracy. These findings demonstrated that despite the large effect size of geography, a universal model based on salivary microbiota has the potential to diagnose caries across the Chinese child population.

## Introduction

Dental caries is a biofilm-mediated, diet modulated, multifactorial, and noncommunicable oral disease (Simón-Soro and Mira, 2015; Pitts et al., 2017), affecting approximately half of children worldwide. If not treated in time, it would affect the mastication function, psychosocial environment and the quality of life of the caries-affected child (Mathur and Dhillon, 2018). Severe caries, an aggressive form of dental caries, can lead to acute pain, sepsis, and potential tooth loss and even interfere with children’s quality of life, nutrition, and school participation (Li et al., 2015). Therefore, preventive measures against caries, as well as improved tools for prognosis early diagnosis, are of particular clinical significance.

Human oral microbiome dysbiosis is increasingly implicated in various local and systemic human diseases, such as dental caries (Yang et al., 2012), gingivitis (Huang et al., 2014), and obesity (Yang et al., 2015). The oral microbial composition depends on many factors (Gupta et al., 2017), including age (Teng et al., 2015), diet (Baroudi et al., 2016), and geography (Gupta et al., 2017). Accumulating evidence supports that changes in oral microbiota continue throughout human life (Ling et al., 2013; Song et al., 2013; Teng et al., 2015; Xu et al., 2015), especially among three dentitions (i.e., deciduous/primary, mixed, and permanent dentition) (Aas et al., 2008; Crielaard et al., 2011). One previous study found that *Prevotella* increased from deciduous, mixed, to permanent dentitions in healthy individuals, and there was a higher proportion of *Proteobacteria* in deciduous dentition than in mixed and permanent dentition (Crielaard et al., 2011). Another study showed that *Lactobacillus* spp. and *Propionibacterium FMA5* were enriched in primary teeth from caries samples, while *Atopobium genomospecies C1* was enriched in permanent teeth (Aas et al., 2008). The mixed dentition stage is a crucial transitional period during which deciduous teeth exfoliate successively and new permanent teeth erupt (Shi et al., 2016). It is not only the main growth and development period of children’s maxillofacial and dental arches but also subject to tremendous changes in host hormones and the immune system (Feres et al., 2016), which may promote maturation of oral microbiota (Feres et al., 2016). Notably, most of the previous microbial studies were focused on early childhood or adult caries (Gross et al., 2010; Ling et al., 2010; Yang et al., 2012; Teng et al., 2015), and there are rare reports on the association of the oral microbiome with health and caries in mixed dentition (Shi et al., 2016; Shi et al., 2018; Xu et al., 2018).

Regarding geographical factors, former studies reported that adult populations from different continental regions or even countries had microbial variations in saliva (Nasidze et al., 2009; Li et al., 2014), and supragingival microbiota differed among ethnic groups (i.e., African American, Burmese, Caucasian, and Hispanic) in children from the same geographic location (i.e., Burma) (Premaraj et al., 2020). Those studies showed the importance of understanding the bacterial community across geography. However, the influence of geographic factors, such as city-scale differences, on the oral microbiome of healthy and diseased children is largely underexplored.

In this study, we address three general questions: (*i*) During the mixed dentition period, do oral communities assemble differently at different host states (i.e., healthy and caries)? (*ii*) How is bacterial diversity partitioned across biogeography, host states and biological sex? (*iii*) Should the geographic factor be taken into account when building classifiers to distinguish children with caries from healthy controls? Here, we conducted a comparison of the saliva microbiome from caries-affected and healthy child cohorts between 6 and 8 years old from two cities in China (Qingdao and Guangzhou) by 16S rRNA gene sequencing. Ecological modeling techniques were further employed to dissect the role of saliva microbiota in caries and geography and probe the predictive value of the microbiome for diagnosing caries by identifying both biogeography- and disease-associated taxa.

## Materials and Methods

### Subject Recruitment and Oral Examination

**Figure 1:**
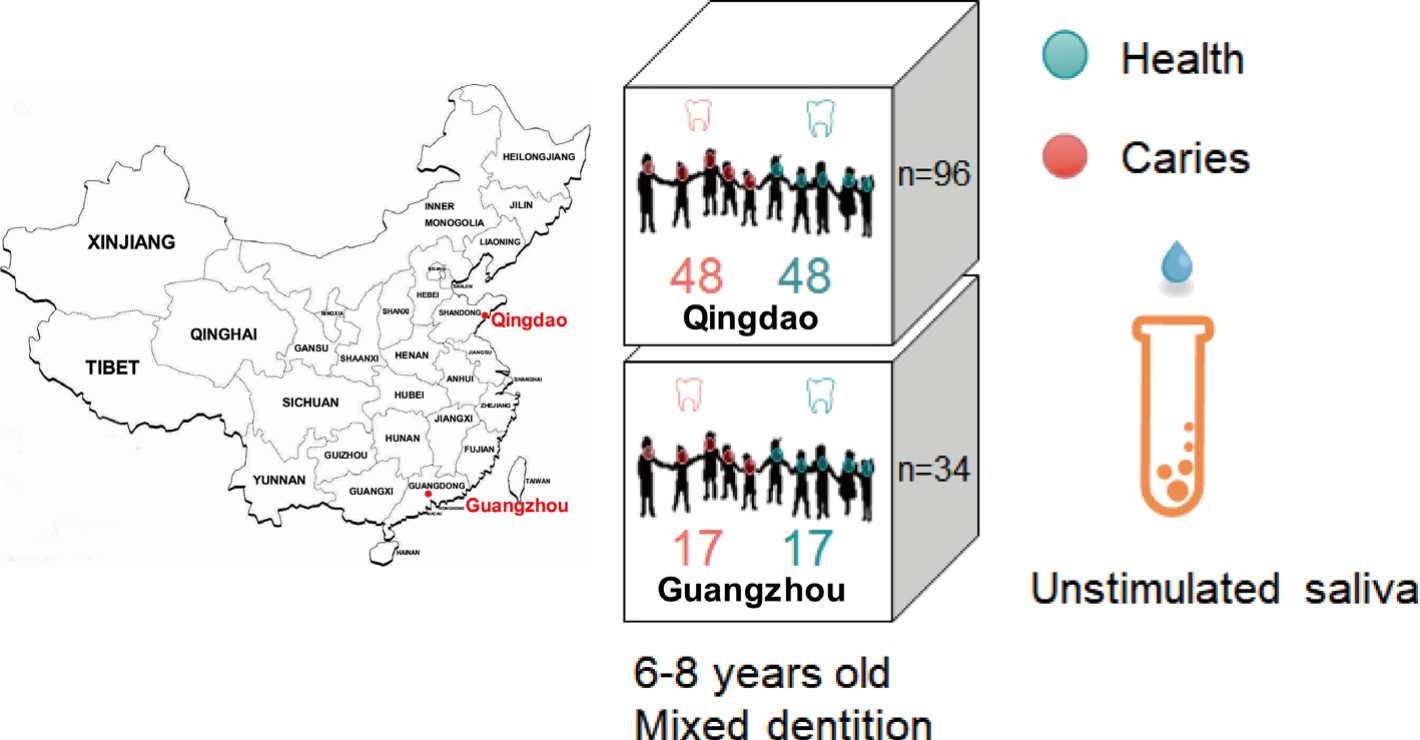
Experimental design that sampled saliva microbiome from caries-affected children and healthy controls in the two Chinese cities of Qingdao and Guangzhou. Unstimulated saliva microbiota from 130 individuals (Qingdao, n=96; Guangzhou, n=34) were compared.

The study was reviewed and approved by the Ethical Committee of Qingdao University (Qingdao, China). Written informed consent was obtained from the legal parents or other guardians of all participants prior to enrollment. The study included two cities in different locations in China: Qingdao is situated in northern China (Shandong Province, **Figure 1**), and Guangzhou is situated in southern China (Guangdong Province, **Figure 1**). Qingdao city has a resident population of 9.50 million and a floating population of 1.61 million, where the dietary habit of residents was relatively high carbohydrate intake preferred (Song and Cho, 2017), such as wheat, steamed bread, noodles, dumplings, etc. Guangzhou is the metropolis of South China with a resident population of 15.3 million and a floating population of 3.5 million, located 1,900 kilometers away from Qingdao. Inhabitants in Guangzhou have more diversity in their diet and prefer a high protein intake as well as rice, soups and desserts. Moreover, Guangzhou has a subtropical monsoon climate with an annual average temperature of 21.8°C, while Qingdao has a monsoon climate of medium latitudes with the characteristics of a marine climate and an annual average temperature of 12.7°C.

All children were from the full-time government primary schools in the urban areas of Qingdao and Guangzhou city with a relatively moderate to high socioeconomic status, where they stayed in all day long, 5 days a week. In addition, we excluded all-girl or all-boy schools. Therefore, the children shared a relatively homogeneous campus living environment within each city. Therefore, these children shared a relatively homogeneous living environment within each city.

All human host volunteers were from an oral health census on the 6-8 years old children (**Figure 1**) from 18 primary schools in Qingdao, China and 11 ones in Guangzhou, China. After oral health survey, 65 healthy and 65 caries-affected children with mixed dentition (see definition below) were chosen for unstimulated saliva sample collection. Oral clinical examinations were performed in their schools by five professional dentists who were previously trained and calibrated for the evaluation and sampling procedures, according to the approach and criteria recommended by the World Health Organization (Yeung, 2014). The intraexaminer reproducibility in both the pilot phase and the main survey was assessed by kappa statistics, which was 0.85. The DMFT (i.e., the number of decayed, missing, and filled teeth-surfaces in permanent dentition) and dmft (i.e., the number of decayed, missing, and filled teeth-surfaces in deciduous dentition) index (Yeung, 2014) was adopted in the current study to measure each children’s caries status and thus to define and distinguish the caries-affected and healthy subjects. The dental examinations were performed using visual-tactile methods by dentists using an intraoral light-emitting diode (LED) light and sterilized examination instruments.

The exclusion criteria of sample collection were as follows: (1) subjects with other oral diseases, such as periodontitis, malocclusion or mucositis; (2) subjects with systematic diseases or congenital diseases, emotional or intellectual disabilities, developmental malformations, or severe infections in other parts of the body; (3) children who refused to cooperate in the dental examination or saliva collection; and (4) antibiotics, probiotics, professionally applied fluorine vanish, or orthodontic appliances were used within the past three months.

### Saliva Collection

After oral clinical examination, 96 (Qingdao) and 34 (Guangzhou) children were chosen for saliva sample collection. Among these, the Qingdao-originated samples were from 48 caries-affected (DMFT+ dmft≧6) and 48 healthy (DMFT+ dmft=0) subjects, while the Guangzhou-originated samples were from 17 caries-affected (DMFT+ dmft ≧6) and 17 healthy (DMFT+ dmft=0) children (**Figure 1**). Children shall avoid eating and drinking water one hour before sampling. Each sampling shall take place between 9:00-12:00 am. Children should keep their head back, eyes closed, and seat upright while taking samples. Subjects were comfortably seated, and after a few minutes of relaxation, they were trained to avoid swallowing saliva and asked to lean forward and spit all the saliva they produced for 5 min into sterile plastic 50 ml tubes. Then, we individually numbered and sealed the samples, placed them in a 4°C sample storage tank, and stored them in a freezer at -80°C for long-term storage.

### DNA Extraction, PCR Amplification, and Sequencing of the Oral Microbiome

Microbial genomic DNA was isolated using lysozyme-containing enzymatic lysis buffer and zirconia-silica beads (BioSpec, Bartlesville, OK) and a DNeasy^®^ Blood and Tissue Kit (Qiagen Valencia, CA). The V1-V3 hypervariable regions of the 16S rRNA gene were subjected to pyrosequencing on 454 Life Sciences Genome Sequencer FLX Titanium (GS-Titanium; 454 Life Sciences, Branford, CT, USA). Amplicons spanning V1-V3 hypervariable region (*Escherichia coli* positions 5-534) of 16S rRNA gene sequence were generated using the forward primer (NNNNNNNTGGAGAGTTTGATCCTGGCTC-AG) and reverse primer (NNNNNNN-TACCGCGGCTGCTGGCAC) incorporating a sample barcode sequence. The PCR conditions were as follows: 2 min initial denaturation at 95°C; 25 cycles of denaturation at 94°C (30 s), annealing at 56°C (25 s), and elongation at 72°C (25 s); and final extension at 72°C for 5 min. The PCR products were separated by 1.2% agarose gel electrophoresis, and the approximately 500 bp fragments were purified using Agencourt AMPure XP (Beckman Coulter, Inc., CA, USA).

Raw sequencing data were processed by using the pipeline tools MOTHUR (Schloss et al., 2009) and QIIME (Caporaso et al., 2010), and pyrosequencing data were analyzed using customized R scripts. The sequences were binned into operational taxonomic units (OTUs) with 97% similarity. OTUs are groups of sequences that are clustered based on similarity, allowing taxonomic assignment.

### Statistical Analysis

Overall, the saliva microbiota was compared in two dimensions: (*i*) between caries-affected samples and healthy controls to discover the potential microbial factors associated with caries and (*ii)* between samples from Qingdao and Guangzhou cities to identify the effect size of geography on saliva microbiota. The Jensen-Shannon distance metric (JSD) and PCoA (Principal Component Analysis) were used to visualize the differential distribution of the between-microbiome difference between sampling groups (e.g., diseased states, city of origin). PERMANOVA analyses were further applied to determine the significance (p-value) and strength (F values) of a given confounding factor in explaining the variation in the oral microbiome. The pairwise p-values from Adonis were corrected for multiple comparisons. To compare the quantitative data in the alpha and beta diversity analysis and biomarker selection, the Kruskal-Wallis rank-sum test was used, and p-values were corrected *via* false discovery rate (FDR) for multiple pairwise comparisons.

### Building the Diagnostic Models of Caries

Random forest (RF) was applied to identify features that are differentially abundant (i.e., present in different abundances) across sample groups and diagnosis models. The N top-ranking caries-discriminatory taxa and geography-discriminatory taxa that led to reasonably good fit were identified based on the ‘rfcv’ function in the random forest package (https://cran.rproject.org/web/packages/randomForest/index.html). RF models were trained to identify disease status in the training set, which included samples from the healthy and caries-affected groups using the taxonomy profiles. The results were evaluated with a 10-fold cross-validation approach, and model performance was evaluated by receiver operating characteristic (ROC) curves. Using the species profiles, the performance of the models based on microbiota was evaluated with a 10-fold cross-validation approach where the original samples were randomly partitioned into 10 groups with a similar distribution of healthy and caries samples. In each cross-validation interaction, nine groups of samples were used as training data and tested samples in the remaining group. The cross-validation process was then repeated 10 times, and per-sample prediction was reported as ones in the test fold. Based on the optimization step that selects the taxonomic level that maximizes model performance, the final RF models were based on the taxonomic profiles at the species level. ROC analysis was then used to evaluate the diagnostic performance of the RF models (https://cran.r-project.org/web/packages/pROC/index.html). In the ROC plots, the x axis represents the true-positive rate (TPR, or sensitivity), and the y axis presents the false-positive rate (FPR, or specificity). The area under the ROC curve (AUC) was calculated to quantify the performance of the RF model.

## Results

### A Geographic View of Oral Microbiota in Dental Caries Children With the Mixed Dentition

To address the three questions above, we compared unstimulated saliva microbiota from 130 individuals aged 6 to 8 years old, representing both healthy children (H group, n=65) and caries-affected children (C group, n=65) from two geographical regions of China: a northern city (Qingdao group) and a southern city (Guangzhou group; **Figure 1**, Materials and Methods). A total of 257,147 post-trimming 16S rRNA gene reads were obtained from all samples, with reads per microbiome numbered 2768 ± 499 on average. In each city, children with DMFT + dmft of zero were designated “Healthy” (“H”); otherwise, they were designated “Caries-affected” (“C”) (DMFT + dmft≧6). The samples in the Qingdao group included 48 caries-affected (DMFT + dmft≧6) and 48 healthy (DMFT + dmft =0) subjects (**Figure 1**, Materials and Methods). For those caries indices in the C group from Qingdao samples, the average dmft values were 8.81 ± 1.10 (deciduous teeth) and the average DMFT values were 1.06 ± 0.93 (permanent teeth), thus the corresponding dmft +DMFT values were 9.70 ± 2.17 in C group on average (**Table S1**), while the dmft/DMFT/dmft +DMFT value was 0 in H group. Guangzhou samples included 17 caries-affected (dmft +DMFT≧6) and 17 healthy (dmft +DMFT =0) children (**Figure 1**, Materials and Methods). Of those C group in Guangzhou samples, the dmft/DMFT/dmft +DMFT values were 8.0 ± 1.27, 0.82 ± 0.63 and 8.82 ± 1.74 correspondingly (**Table S2**), while the dmft/DMFT/dmft +DMFT value was 0 in H group. First, we compared the healthy and caries-affected saliva microbiomes in the Qingdao group to explore whether oral communities assemble differently in different host states. Second, the impact of the various factors on the oral microbiota in our pediatric study was assessed. Last, we applied multiple strategies to construct and optimize the caries diagnosis model to clarify the impact of geography on the oral microbiome.

### Dental Caries Altered Saliva Microbiota in the Mixed Dentition

**Figure 2:**
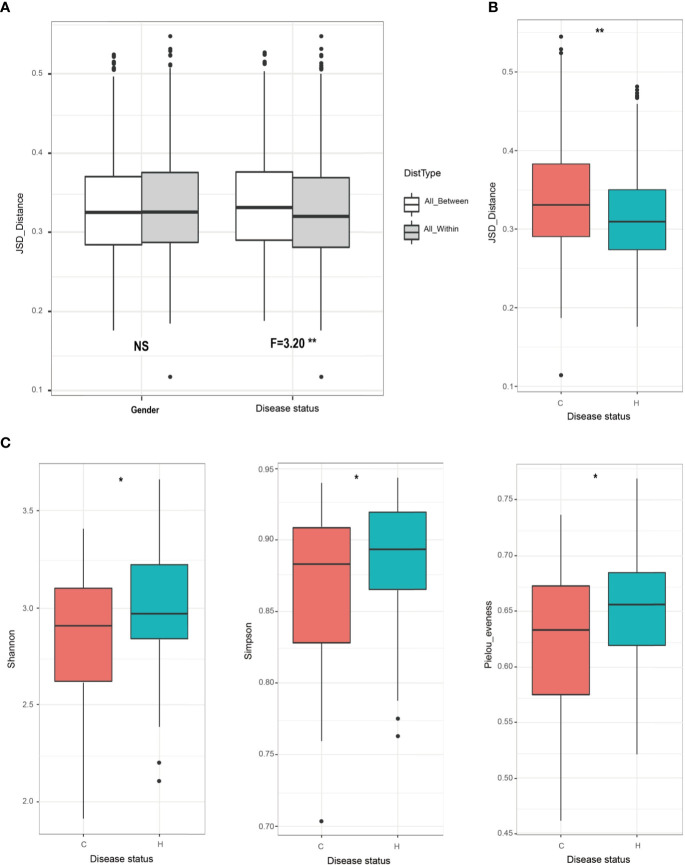
Oral microbial diversity comparisons between caries and healthy children in Qingdao cohort. **(A)** Salivary microbiota variation was compared within and between disease status (i.e., H or C), or gender based on the Jensen-Shannon distances. Only disease status exhibits a strong effect on the composition of saliva microbiota (F=3.20**). **(B)** Caries-free children have more conservative microbiota than do children with caries (**p<0.01). **(C)** Alpha diversity comparisons between the C and H groups using on Shannon, Simpson, and Pielou’s evenness index. Indices showed that, the alpha-diversity values from the caries-affected samples were significantly decreased than those in healthy samples (Shannon, *p=0.041; Simpson, *p=0.046; Pielou’s evenness, *p=0.02).

To investigate whether and how caries affects oral microbiota in the mixed dentition stage, we first compared beta diversity within and between disease status (i.e., health and caries-affected) and gender based on the Jensen-Shannon distances and PCoA based on the Qingdao samples. We found that disease status exhibited a remarkable effect on shaping salivary microbiota (*p<*0.01, F=3.20) rather than gender (*p*>0.05; **Figure 2A**). Furthermore, the C group exhibited significant variability, while the H group was relatively conserved in microbial community structure (*p*<0.05; **Figure 2B**). Additionally, the H and C groups showed separation trend in the chart plotted based on principal coordinate analysis (**Figure S1**). Next, we assessed the impact of the disease status on the alpha diversity represented by Shannon, Simpson, and Pielou’s evenness indices. The results showed that the alpha diversity was significantly lower in the C group than in the H group (all *p<*0.01; **Figure 2C**). Finally, we quantitatively profiled the bacterial taxa from the phylum to species level to characterize the mixed-dentition microbial composition (**Figure S2A** **)** and then tested whether there were any caries-enriched and caries-depleted taxa. All sequences were distributed in 13 bacterial phyla that included six predominant phyla (accounting for > 99% of the microbial diversity; **Figure S2A**), namely, *Firmicutes* (78.0%), *Actinobacteria* (11.9%), *Bacteroidetes* (5.0%), *TM7* (2.0%), *Proteobacteria* (1.6%) and *Fusobacteria* (1.4%). At the genus level, a total of 124 genera were identified, among which the most frequently detected genera (the four most abundant genera that each represented at least 5% in the average relative abundance) were *Streptococcus* (51.4%), *Gemella* (11.2%), *Actinomyces* (8.7%) and *Granulicatella* (5.8%); **Figure S2A**). Moreover, no ‘caries-specific’ taxon (present in one status but absent in the other) was detected between the two groups. At the genus level, *Lactobacillus*, *Gemella*, *Cryptobacterium* and *Mitsuokella* were found to have significantly higher relative abundances in the C group, while *Leptotrichia*, *Porphyromonas*, *Peptococcus*, *TM7*, and *Tannerella* were higher in the H group (all *p*<0.05, **Figure S2B**). At the species level, *Actinomyces IP073*, *Lactobacillus gasseri*, *Prevotella denticola*, *Propionibacterium FMA5*, *Streptococcus anginosus*, *Streptococcus mutans*, *Streptococcus sobrinus* and *Actinomyces gerencseriae* were found to have significantly higher relative abundances in the C group, while *Porphyromonas cato*niae, *Porphyromonas CW034*, *Propionibacterium propionicum*, *Tannerella oral taxon 808*, *TM7 oral taxon 352* and *uncultured Lachnospiraceae oral taxon 100* were higher in the H group (all *p*<0.05, **Figure S2C**).

### Geography Affected the Saliva Microbiota More Than Caries Status

**Figure 3:**
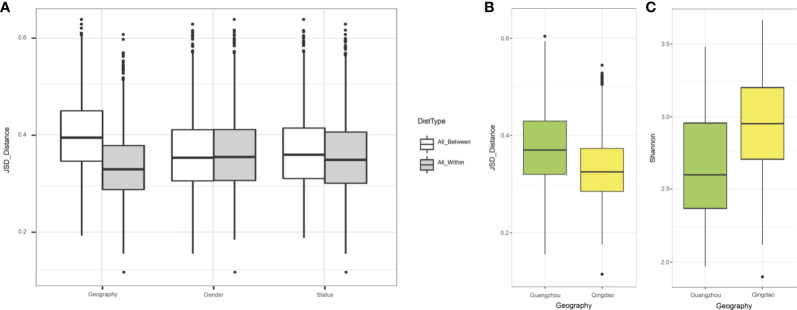
The remarkable impact of city of origin on oral microbiomes. **(A)** The effect size of geography, gender and host’s disease status on saliva microbiota based on Jensen-Shannon distance. The city of origin exhibited the strongest effect on bacterial composition of the saliva microbiome, followed by host status and gender factor. **(B)** Beta-diversity difference between Qingdao and Guangzhou groups measured by JSD distances. **(C)** Alpha diversity difference between Qingdao and Guangzhou groups measured by Shannon index.

To elucidate the impact of geography on the oral microbiota, we included a second group of 34 age-matched children (17 with caries-affected and 17 healthy subjects) from the southern city in China (Guangzhou group), approximately 1900 kilometers southwest of the northern city (Qingdao group; **Materials and Methods**). The analyses over the two cities showed that geography exhibited a higher effect on defining microbiota composition (*p*<0.001) than did caries status (*p*<0.05), and the two factors jointly explained up to 54% of the variation in microbiota, suggesting that they were the major factors shaping oral microbiota (**Figure 3A**). Besides, the Qingdao and Guangzhou groups showed separation trend in the chart plotted based on principal coordinate analysis (**Figure S3**). Furthermore, the Qingdao microbiota communities were more similar to each other than the Guangzhou microbiota: Guangzhou city samples showed higher within-group variability in Jensen-Shannon metrics (**Figure 3B**; *p*<0.05), and Qingdao city samples were significantly more diverse in Shannon indices than that in Guangzhou (**Figure 3C**; *p*<0.05).

**Figure 4:**
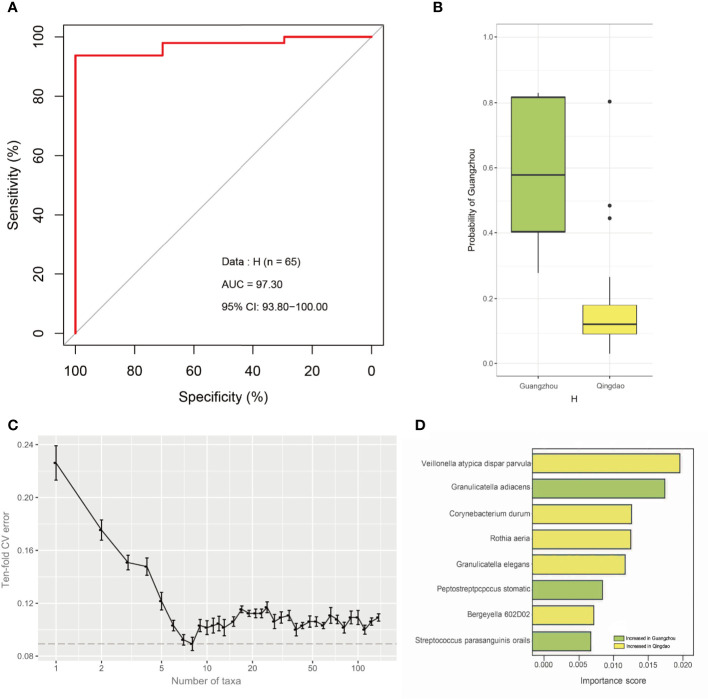
The strong geographical background of the healthy oral microbiota and key drivers. **(A)** Microbiome can classify the city of origin of healthy samples with a high accuracy. We utilized the Random Forests machine learning algorithm to quantify the city-associated difference in the saliva microbiota from healthy population. City (Qingdao and Guangzhou, China) can be distinguished with high AUC in the random forests model. And the healthy microbiota can predict city-origin with high accuracy (AUC=97.30%). **(B)** Box plot indicates the prediction probability of Guangzhou city in healthy samples. The probability of Guangzhou was significantly higher in the Guangzhou samples than in the Qingdao samples in H group. **(C)** Relationship between the numbers of variables used in the reduced models and the corresponding predictive performance (the error bar denotes SD). **(D)** The importance score of eight the most discriminating species in the diagnosis model to predict city origin. The bar length at each row indicates relative contribution of the species to the RF model.

To identify geography-specific markers contributing to predicting city origins, we first built classification models *via* the random forest (RF) machine learning algorithm using healthy samples as the training set. The city origin was predicted from healthy samples with 78.88% accuracy (area under the concentration curve [AUC]: 97.30%; CI: 93.80%-100.00%, **Figure 4A**). The probability of Guangzhou city was significantly higher in Guangzhou city samples than in Qingdao city samples from the H group (Wilcoxon test, *p*<0.05, **Figure 4B**). Next, the RF model ranked the contribution of each predictor based on the variable importance, where we can identify the most discriminatory bacteria between two cities. Performance improvement was minimal when the top eight most discriminatory species were included (**Figure 4C** **).** Eight geography-specific marker species underlying the power of the healthy model were identified, namely, *Veillonella atypica/dispar/parvula*, *Granulicatella elegans*, *Corynebacterium durum*, *Rothia aeria, Bergeyella 602D02*, *Granulicatella adiacens, Peptostreptococcus stomatis* and *Streptococcus parasanguinis oralis* (**Figure 4D** **)**. Among them, the relative abundance of the former five species was higher in Qingdao city samples than in the Guangzhou city samples (Wilcoxon test, adjusted *p*<0.05), while that of the latter three taxa significantly increased in Qingdao city samples (Wilcoxon test, adjusted *p*<0.05, **Figure S4A**). Moreover, these taxa were shared in caries samples, representing 12.88% and 13.13% abundance for healthy and caries samples, respectively. Finally, application of the eight-marker-based model on the caries samples resulted in 92.31% accuracy (AUC: 95.00%; **Figure S4B**), and the probability of Guangzhou city was significantly higher in the Guangzhou samples than in the Qingdao samples from the C group (Wilcoxon test, *p*<0.05; **Figure S4C**). Thus, geography-specific differences in the salivary microbiome were consistent, irrespective of health status.

### A Universal Disease Diagnosis Model for All Samples Across Geographic Locations

Consistent with the results for the Qingdao city samples, a reduction in alpha diversity was associated with caries (*p*<0.05; Shannon index; **Figure S5A**) in all samples from both cities, and the beta diversity was distinct between caries and healthy microbiota (*p*<0.05, F=1.00; **Figure S5B**). These results suggested the feasibility of caries diagnosis based on oral microbiota in different geographic locations.

**Figure 5:**
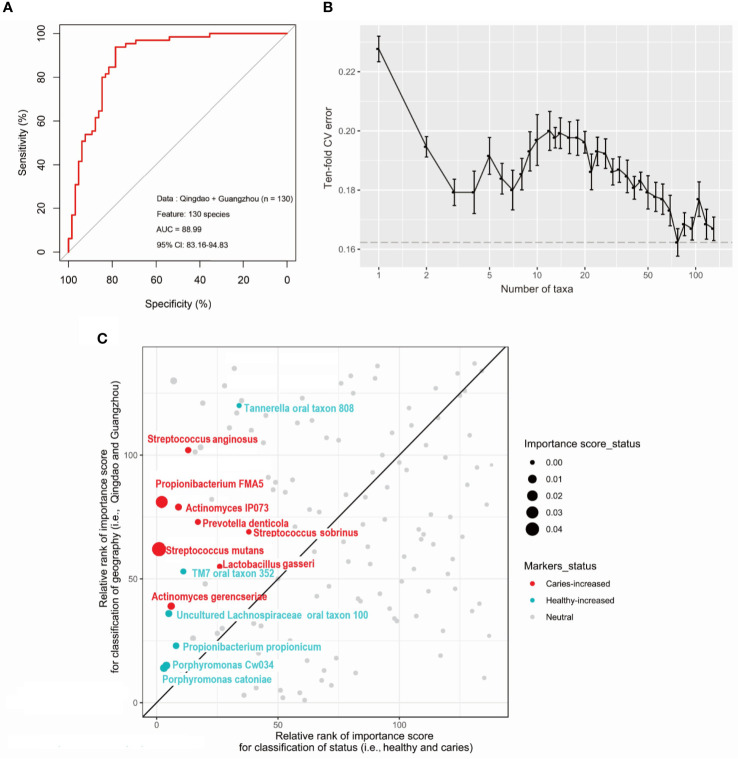
Caries diagnostic models based on oral microbiome detrended for geography. We constructed caries diagnostic models using 130 oral microbial species from both Qingdao and Guangzhou populations. **(A)** Saliva microbiota can predict caries status with a remarkably high accuracy (AUC=88.99%). **(B)** The relationship between the numbers of variables used in the reduced Random Forest model and the corresponding predictive performance (the error bar denotes SD). **(C)** The most caries-discriminatory taxa (N=14) do not correlate with geography. The scatterplot shows the relative rank of microbial markers in both Random Forest models for classifying disease status and geographic locations. Any dots on the reference line which slope=1 suggests a taxon is equally important to both disease states and geography.

**Figure 6:**
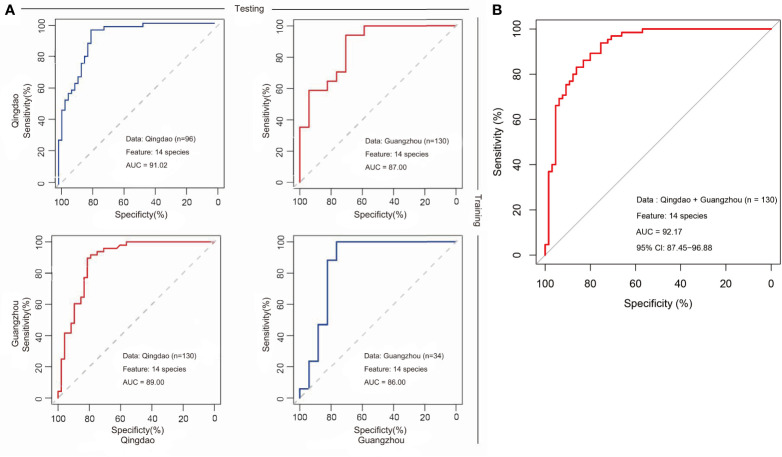
Cross-applications of caries diagnosis models based on microbiomes from Qingdao and Guangzhou cohorts. We constructed caries diagnosis models in the saliva microbiota from either Qingdao or Guangzhou population. Using either Qingdao or Guangzhou microbiota, status (heathy and caries) can be distinguished with a high prediction accuracy (AUC) in the 10-fold CV for random forests model. **(A)** The prediction performance of models in the Qingdao (AUC=91.02%, Guangzhou (AUC=86.16%) and model application from one city to another. A classification model trained in Qingdao data and tested in Guangzhou Data resulting in a AUC=87.00%; A classification model trained in Guangzhou data and tested in Qingdao Data resulting in a AUC=89.00%. **(B)** The predictive performance using data from two cities (AUC=92.17%).

There were three strategies to construct and optimize the caries diagnosis model. First, to test the effect of taxonomic level on the discriminatory power of the RF model, the models were constructed based on taxa at the phylum, genus, and species levels to discriminate between healthy and caries samples using two city datasets. We found that the use of species-level taxa maximized (AUC: 88.56%, CI: ﻿83.56%−94.61%) compared with that of the others at the phylum (AUC: 64.11%, CI: 54.54%−73.67%) and genus (AUC:77.61%, CI: ﻿69.48%−85.74%) levels (**Figure S6**). Second, to test whether differences in oral microbiota in caries were consistent by city, we built RF models in each city (i.e., Qingdao and Guangzhou) and achieved diagnosis accuracies of 84.38% and 76.47%. Furthermore, training a diagnosis model in one dataset and applying it to another led to lower yet still decent and meaningful performance (**Figure S7**). Specifically, application of the Qingdao model (i.e., the Qingdao cohort as training data) on the Guangzhou dataset led to a reduction in the AUC from 91.10% to 83.00%, and similarly, application of the Guangzhou model (i.e., the Guangzhou cohort as training data) on the Qingdao dataset led to a reduction in the AUC from 85.81% to 80.00% (**Figure S7**). Third, we built RF models using all caries and healthy samples from the two geographic locations. Unexpectedly, excluding eight geography-specific signatures from the species profile rarely affected the classification performance, with AUCs from 88.56% to 88.99% (**Figure 5A** **)**. Moreover, intriguingly, these most discriminatory taxa associated with caries state did not show correlation with geography in the healthy samples (**Figure 5C**) and vice versa (**Figure S8**) in either the geographic or caries diagnosis model. Underlying the power of the model using the species-level profile that ruled out these geographic signatures, fourteen bacterial species markers were identified based on both the rank order of important scores (**Figures 5B, C** **)** and the Wilcoxon test results (adjusted *p*<0.05; **Figure S1C**). Among them, eight taxa (i.e., *Streptococcus mutans*, *Actinomyces gerencseriae*, *Propionibacterium FMA5*, *Actinomyces IP073*, *Streptococcus anginosus*, *Lactobacillus gasseri*, *Streptococcus sobrinus*, and *Prevotella denticola*) were caries-enriched, while the other six taxa (i.e., *Tannerella oral taxon 808*, *Propionibacterium propionicum*, *Uncultured Lachnospiraceae oral taxon 100*, *Porphyromonas CW034*, *Porphyromonas catoniae* and *TM7 oral taxon 352*) were caries-depleted (adjusted *p*<0.05; **Figure S1C**). Consequently, we constructed the final caries diagnosis model based on the fourteen species selected, which led to an increase in predictive performance in Qingdao city (AUC: 91.02%; CI: 85.27%-96.76%; **Figure 6A**), Guangzhou city (AUC: 86.16%; CI: 71.57%-100.00%; **Figure 6A**) and across two cities (AUC: 92.17%; CI: 87.45%-96.88%; **Figure 6B**). Notably, *Streptococcus mutans* (*S. mutans*) with the top importance score in the model (Wilcoxon test, adjusted *p*<0.05, **Figure 5C** and **Figure S2C**) has previously been documented to play a critical role in caries pathogenesis. Using only *S. mutans* as a predictor, the simplified random forest model led to a lower yet decent performance (AUC=81.62%, CI: 74.40%-88.84%, **Figure S9**). However, *S. mutans* was not detected in all of the samples (the occurrence rate in the caries sample=78.5%, the occurrence rate in the healthy sample=30.8%), as well as the others (**Figure S10**), suggesting that dental caries is not associated with a single taxon but in fact with a complex community.

## Discussion

It has been well documented that in dental caries, environmental perturbation alters the balance of the oral microbiota and eventually leads to a predominance of cariogenic bacteria, resulting in sustained demineralization of tooth hard tissue (Kidd and Fejerskov, 2013). Evidence has recently emerged that the oral microbiome may depend on age, oral dentition, diet and geography (Crielaard et al., 2011; Teng et al., 2015; Hansen et al., 2018; Dashper et al., 2019; Mark Welch et al., 2020). Effectively reducing dental caries burden requires a better understanding of its determinants. To address this issue, we profiled and compared saliva microbiota from 130 individuals aged 6 to 8 years old, representing both caries-affected and healthy control children from two geographical regions of China: a northern city (Qingdao) and a southern one (Guangzhou).

First, we characterized the dysbiotic saliva microbiome in caries in human populations in terms of alpha diversity, beta diversity and bacterial composition. Similar to our observations, previous studies of various age stages of individuals have shown that caries status favored reduced microbial diversity (Li et al., 2007; Teng et al., 2015; de Jesus et al., 2020). Such a reduction in alpha diversity is likely caused by increased carbohydrate consumption and fermentation, leading to acid production and secretion. The low-pH environment probably selects acidogenic and aciduric taxa, which could thrive under the condition (Marsh, 2006). The beta-diversity analysis showed that saliva microbial communities significantly differed between diseased and healthy children. Moreover, caries children also have higher Jensen-Shannon distances than healthy children. This is likely because caries microbiomes have higher intro-group variation and more personalized microbiomes than healthy microbiomes, which are more similar to each other (Yang et al., 2012). Moreover, our data substantiate existing evidence that organisms other than *Streptococcus mutans* and *Lactobacilli* play a role in the development and progression of dental caries. At the genus level, the caries microbiome harbored a higher abundance of *Lactobacillus, Gemella* and *Cryptobacterium* than healthy controls, which is in line with previous studies (Jiang et al., 2014; Ma et al., 2015; Ledder et al., 2018). At the species level, the increase in *non-mutans streptococci* (i.e., *S. anginosus* and *S. sobrinus*) and *Actinomyces_gerencseriae* in the C group was not surprising. They were recognized as acidogenic and aciduric bacteria, which have been reported to produce weaker acid resulting in caries initiation and thrive during caries progression in low pH conditions (e.g., pH=5.0) (Svensäter et al., 2003; Takahashi and Nyvad, 2011; Xu et al., 2018). Similarly, according to our and other studies (Yang et al., 2012), *Prevotella denticola* was significantly enriched in caries and was identified as the main predictor of caries, which potentially have proteolytic/amino acid-degrading activities. *Propionibacterium FMA5* was implicated in dental caries from young permanent teeth (Gross et al., 2010)and root caries from elderly individuals (Preza et al., 2008). In addition, *S. mutans* was identified in relatively low abundance, and the detection rate was relatively low (AUC=81.62%). Consistently, previous studies found that despite a significant enrichment of *S. mutans* with caries development, several bacteria were far more abundant in the carious lesions (Simón-Soro et al., 2013). Our findings illustrated that dental caries in the mixed dentition resulted from widespread shifts in the oral microbial community instead of any particular taxa from healthy to diseased status, supporting the “ecological plaque hypothesis” (Marsh, 2003; Takahashi and Nyvad, 2011).

Next, our data included children from two cities of China: Qingdao (N group) and Guangzhou (S group), between which the distance was approximately 1900 kilometers. We found that alpha and beta diversity in background oral microbiomes are radically distinct across geographic locations. Thus, geography accounted for the highest variance in the salivary bacterial profiles compared to other confounding factors, such as caries state or host gender. In previous studies, saliva microbial profiles can vary greatly across large-scale geographic locations (e.g., the continental region (Nasidze et al., 2009) or country (Li et al., 2014) or by ethnicity within one nation (Mason et al., 2013). However, few studies to date have systematically investigated the oral microbiome from the mixed dentition of Chinese subjects residing in different cities. This makes it challenging to directly compare caries microbiomes across studies and test the generalizability of microbiome-based diagnosis models across geography. Geography is a considerable yet complex factor influencing the development of microbiome-based diagnostic models. First, diet can contribute greatly to geographic differences across population (Song and Cho, 2017). The diet in Qingdao city typically encompasses a wider variety of carbohydrates than that in Guangzhou city, and fatty foodstuffs may supply a more complex array of substrates and allow more diverse bacterial species to thrive in the oral cavity (Li et al., 2014). Additionally, these unique food nutrients have an indispensable effect on the microbial ecology of dental caries (DiNicolantonio and O’Keefe, 2018). Second, the population composition in cities may largely determine intraindividual microbiome variation. With the rapid economic development in Guangzhou, an increasing number of citizens from other parts of China have migrated to well-developed southern cities to seek job opportunities, which has resulted in higher population-level diversity in Guangzhou city. In contrast, those in Qingdao city reflected a more homogenous group from a relatively restricted area. This might be a plausible explanation why we observed a higher interindividual microbiome diversity in Guangzhou’s population. Although the mechanism for the city-dependent microbiome remains obscure, host genetics, climate, dietary patterns, built environments (Chase et al., 2016) and other epidemiological factors should be further considered in developing a microbiome-based diagnostic model of ECC (Gupta et al., 2017).

Finally, we built a universal classification to diagnose caries using oral bacterial species by appropriately detrending the geographic effect in microbiome data. Despite the considerable differences between the two cohorts, a caries diagnosis model built from a single city can still be applied across the two cities with decent accuracy. Moreover, although geographic factors showed a larger effect size in defining oral microbiome data than caries state, city-specific markers had little impact on the prediction performance of caries classification models. Intriguingly, disease-specific biomarkers showed no correlation with geography. These results suggested the feasibility of universal caries diagnosis independent of geographic distances among populations. As a result, the caries diagnosis model consisting of the top 14 bacterial oral species can reliably diagnose caries with 83.08% accuracy (AUC=92.17%) across cities. Our previous and other studies have verified the diagnostic and predictive efficacy of oral microbiota using random forest classification models in deciduous and permanent dentition (Huang et al., 2014; Teng et al., 2015; Hemadi et al., 2017; Wang et al., 2019). Together, these results suggested that caries diagnosis models were biogeography-independent using saliva microbial profiles.

There were several limitations in the current study, and different factors might affect the results. For example, (*i*) the sample size of Guangzhou city here is relatively small, which should be increased to allow a better statistical comparison of the microbial diversity with Qingdao (n=96) samples. (*ii*) Cross−sectional data to examine the link between disease status and geography with dental caries are relatively limited. To further test the causal relationship, a longitudinal design should be conducted. (*iii*) Whether universal diagnosis models are generally applicable in other cities is not yet clear, and more environments and geographic regions must be observed. Future efforts tackling these questions are key to more precise dental caries therapies.

## Conclusions

To our knowledge, this is the first study to use current molecular techniques to the differences between the bacterial composition of the saliva microbiota in mixed dentitions of caries-affected and healthy children living in different geographic locations: either Qingdao or Guangzhou of China. Using machine learning approaches, we also revealed that although geography has the most remarkable effect size on salivary microbiota (the saliva microbiome can predict the originated city with near 100% accuracy), a universal model based on fourteen bacterial species can diagnose caries with 83.08% accuracy across cities (area under the concentration-time curve [AUC], 92.17%). Our study underscores the possibility of employing saliva microbiota for a universal diagnosis method, which can be probed for other dentition stages of oral caries and for caries in other geographic locations.

## Data Availability Statement

The datasets presented in this study can be found in online repositories. The names of the repository/repositories and accession number(s) can be found below: Microbiome Search Engine - http://mse.single-cell.cn/index.php/mse/get_by_project/P_SCC0006.

## Ethics Statement

The studies involving human participants were reviewed and approved by Ethical Committee of Qingdao University (Qingdao, China). Written informed consent to participate in this study was provided by the participants’ legal guardian/next of kin.

## Author Contributions

The study was designed by SL, SH, FY, ZC, QG, YT, and FT. SL, LZ, YZ, and FL performed clinical examination and sample collection. KT and JL performed sample processing. SH, FT, and YG contributed to statistical analysis methods. SL, FY, and FT wrote the paper. All authors contributed to the article and approved the submitted version.

## Funding

Our work was supported in part by grants 81670979 and 31600099 from the Natural Science Foundation of China. The funders had no role in the study design, data collection and analysis, decision to publish, or preparation of the manuscript.

## Conflict of Interest

The authors declare that the research was conducted in the absence of any commercial or financial relationships that could be construed as a potential conflict of interest.
