# Supplementary material for: Geographic Variation Did Not Affect the Predictive Power of Salivary Microbiota for Caries in Children With Mixed Dentition
Source: Front Cell Infect Microbiol. 2021 Jun 18;11:680288. doi: 10.3389/fcimb.2021.680288 (PMC8250437; doi:10.3389/fcimb.2021.680288)
Supplement: Supplementary file 1 [file DataSheet_1.docx]

**Supplementary Figures and Tables**

*
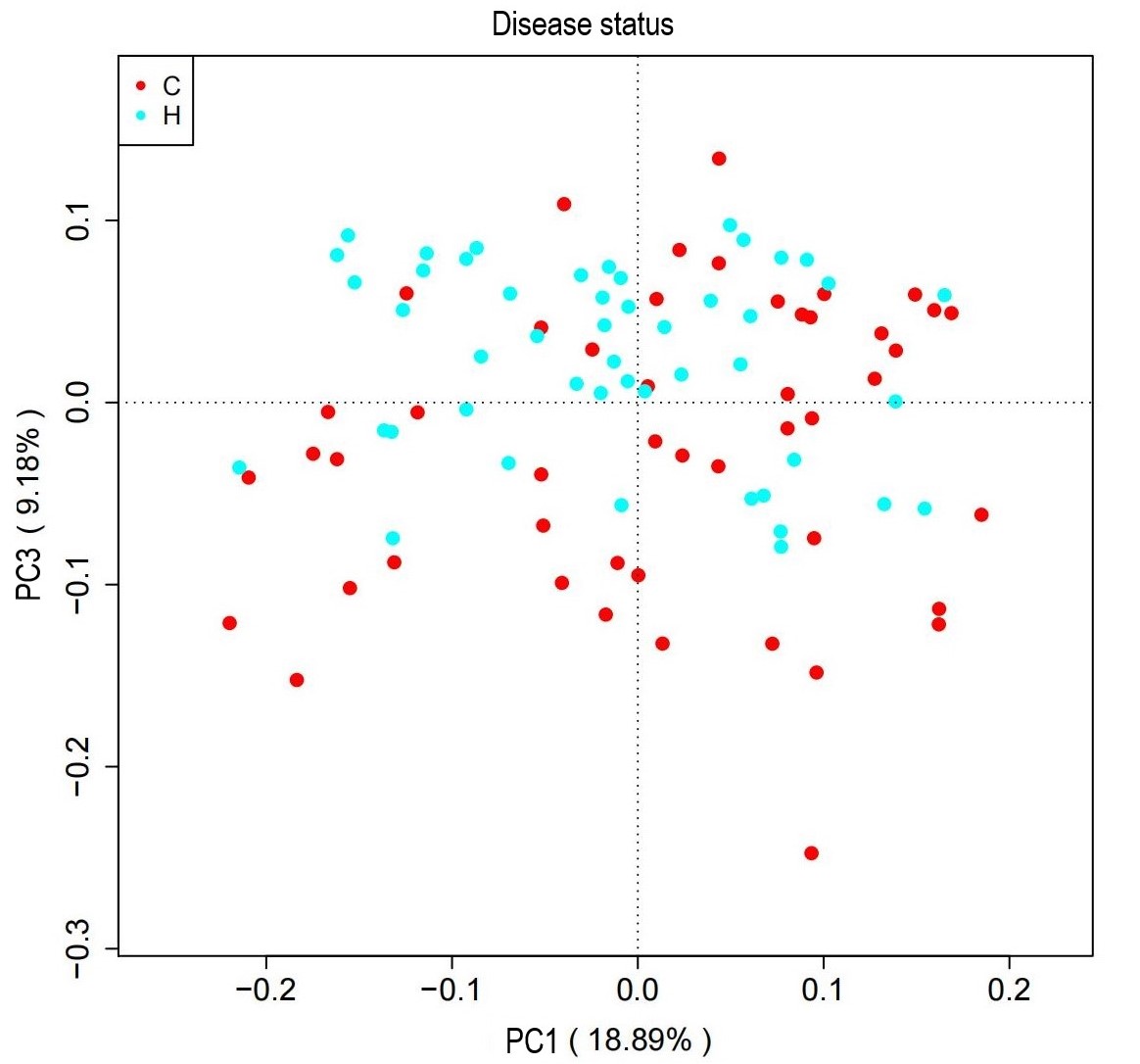
*

**Supplementary Figure 1. Microbial community variation within healthy and caries-affected groups using Jensen-Shannon distances** Communities clustered using PCoA of Jensen-Shannon distance matrix from 96 samples in Qingdao city. Each point corresponds to a sample colored by disese status. Blue dots: heathy samples; read dots: caries-affected samples


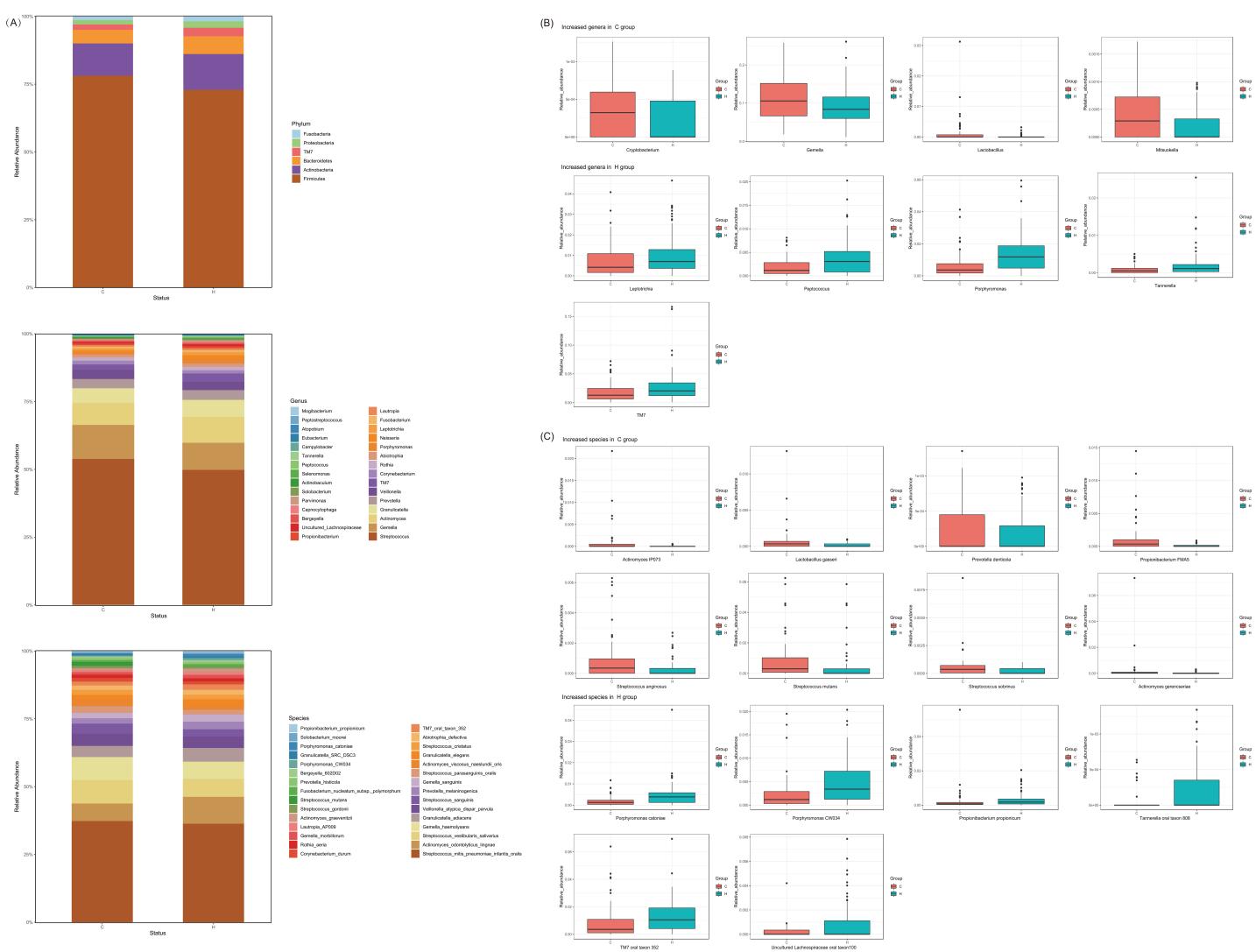


**Supplementary Figure 2. The bar plots show the relative abundance of bacterial taxa between healthy and caries-affected children in Qingdao city, China.** (A) Relative abundances of microbes at the phylum, genus, and species levels between the H and C groups. (B) Relative abundances of microbes at the genus level between the H and C groups. (C) Relative abundances of microbes at the species level between the H and C groups.


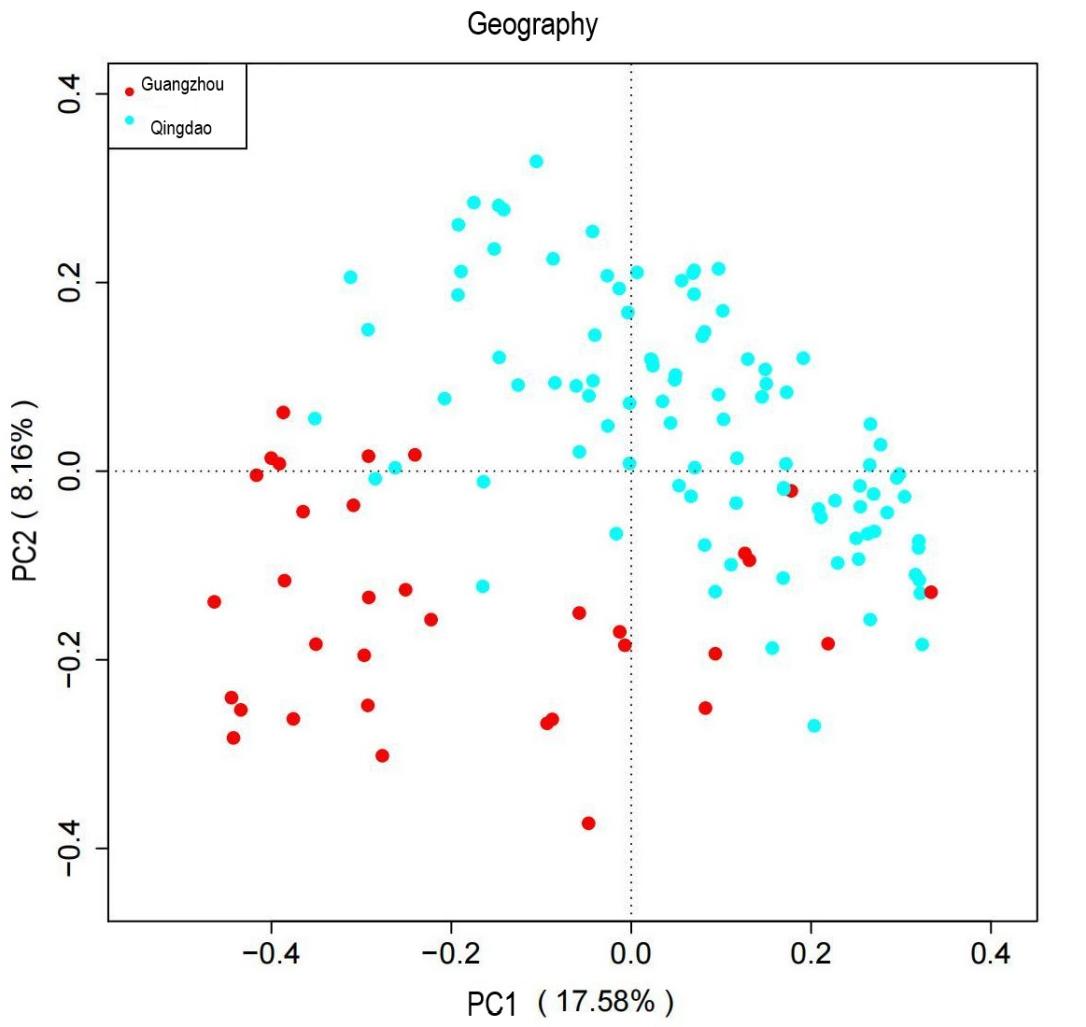


**Supplementary Figure 3. The geographic factor shaping the oral bacterial community composition in all samples.** The Qingdao and Guangzhou group showed a relatively significant separation in the chart plotted based on principal coordinate analysis. Communities clustered using PCoA of Jensen-Shannon distance matrix from 130 samples from both the Qingdao and Guangzhou group. Each point corresponds to a sample colored by city. Blue dots: Qingdao samples; read dots: Guangzhou samples.


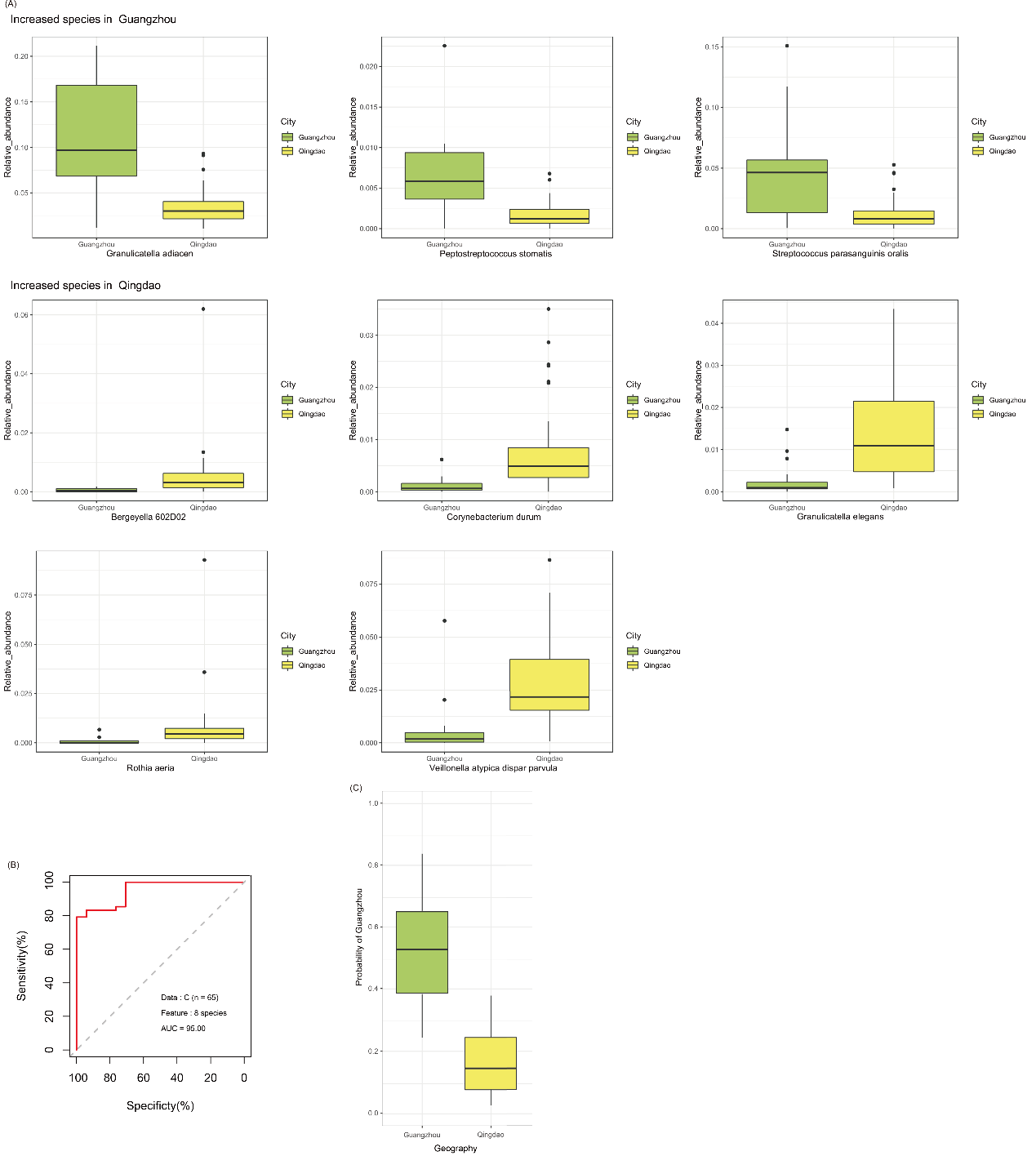


**Supplementary Figure 4. Geography-associated oral microbiome.** (**A**) Boxplots indicate the relative abundance of the 8 species between Qingdao and Guangzhou group. (**B**) A classification model classified the city of origin based on 8 key microbial drivers from hosts in the C group. (**C**) Box plot shows the prediction probability of Guangzhou city in caries samples. The probability of Guangzhou city was significantly higher in the Guangzhou samples than in the Qingdao samples from the C group.


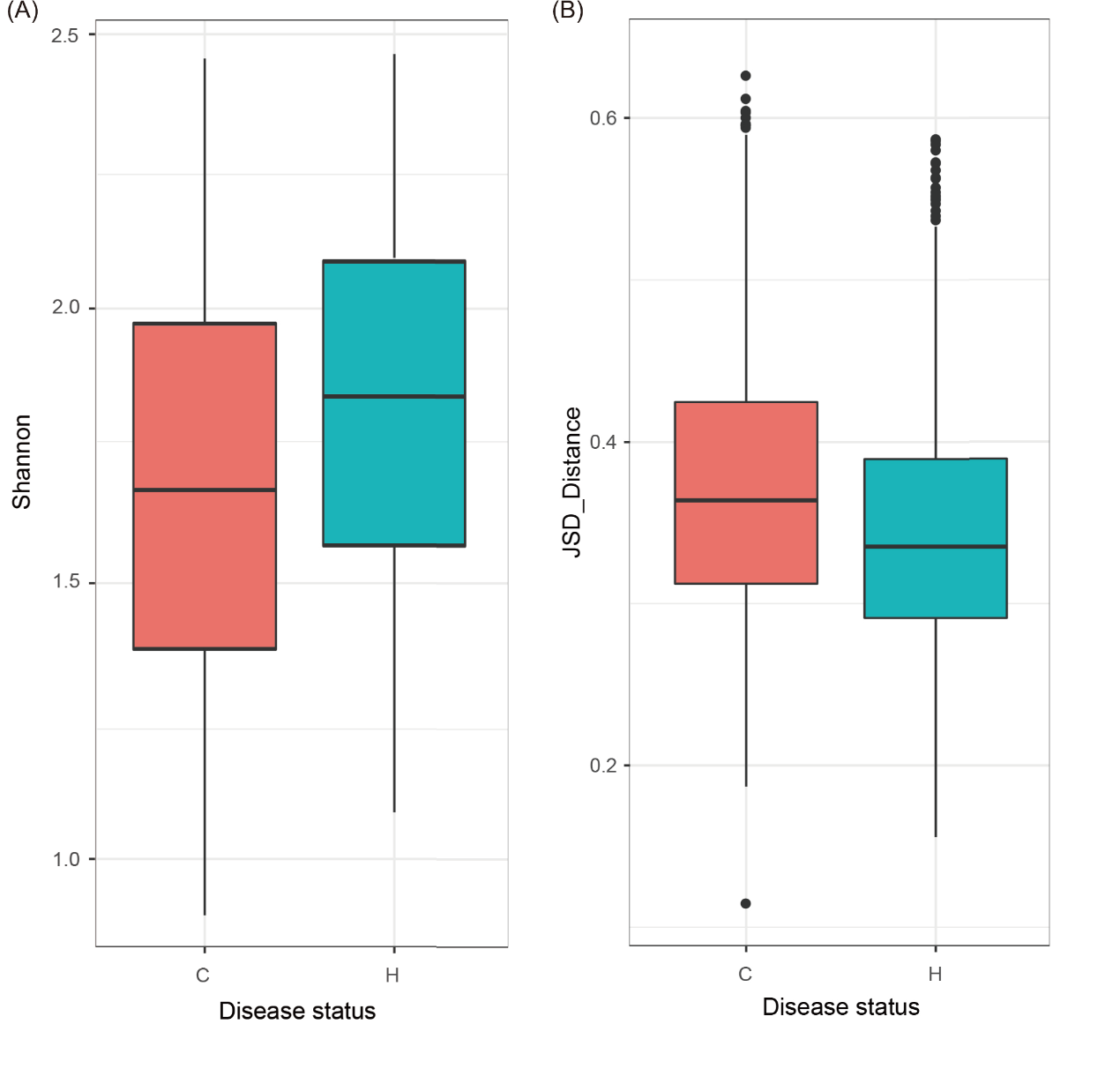


**Supplementary Figure 5. Alpha and beta diversity of all samples in term of disease status.** (**A**) Caries samples featured a reduction in alpha diversity. (**B**) The beta diversity measured by Jensen-Shannon distance was distinctive between caries and healthy microbiota.


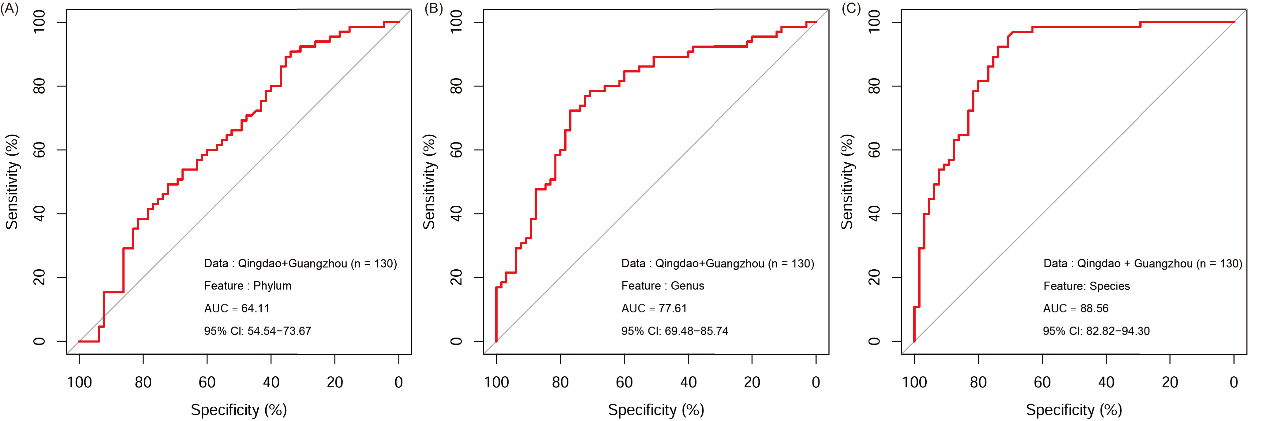


**Supplementary Figure 6. Classification models based on taxa at the phylum (A), genus (B), and species (C) levels to discriminate between healthy and caries samples from all geographic locations**. The prediction accuracy (AUC) was maximized at the species level. The AUC of all samples-based Random Forests classifier of caries diagnosis is 64.11,77.61 and 88.56 respectively.


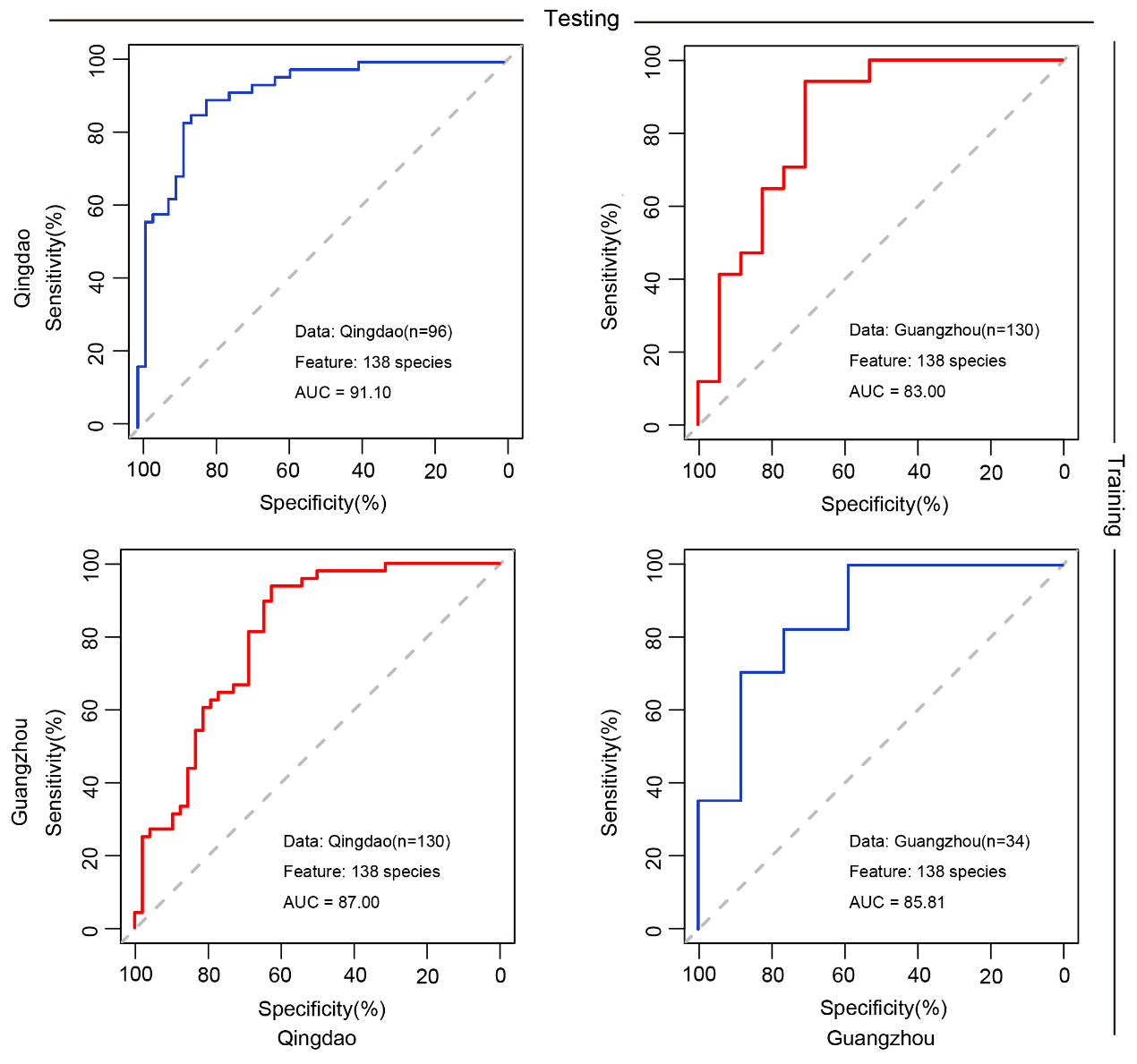


**Supplementary Figure 7. The performance of cross-city prediction using each city-specific caries diagnostic models.** The blue lines indicate the ROC curves evaluating the caries classification model self-validated in each city. The red lines indicate the ROC curves evaluating the application of a caries classification model from one city to another. The performance of the models and their applications to the other dataset was scored by AUC in the ROC analysis.


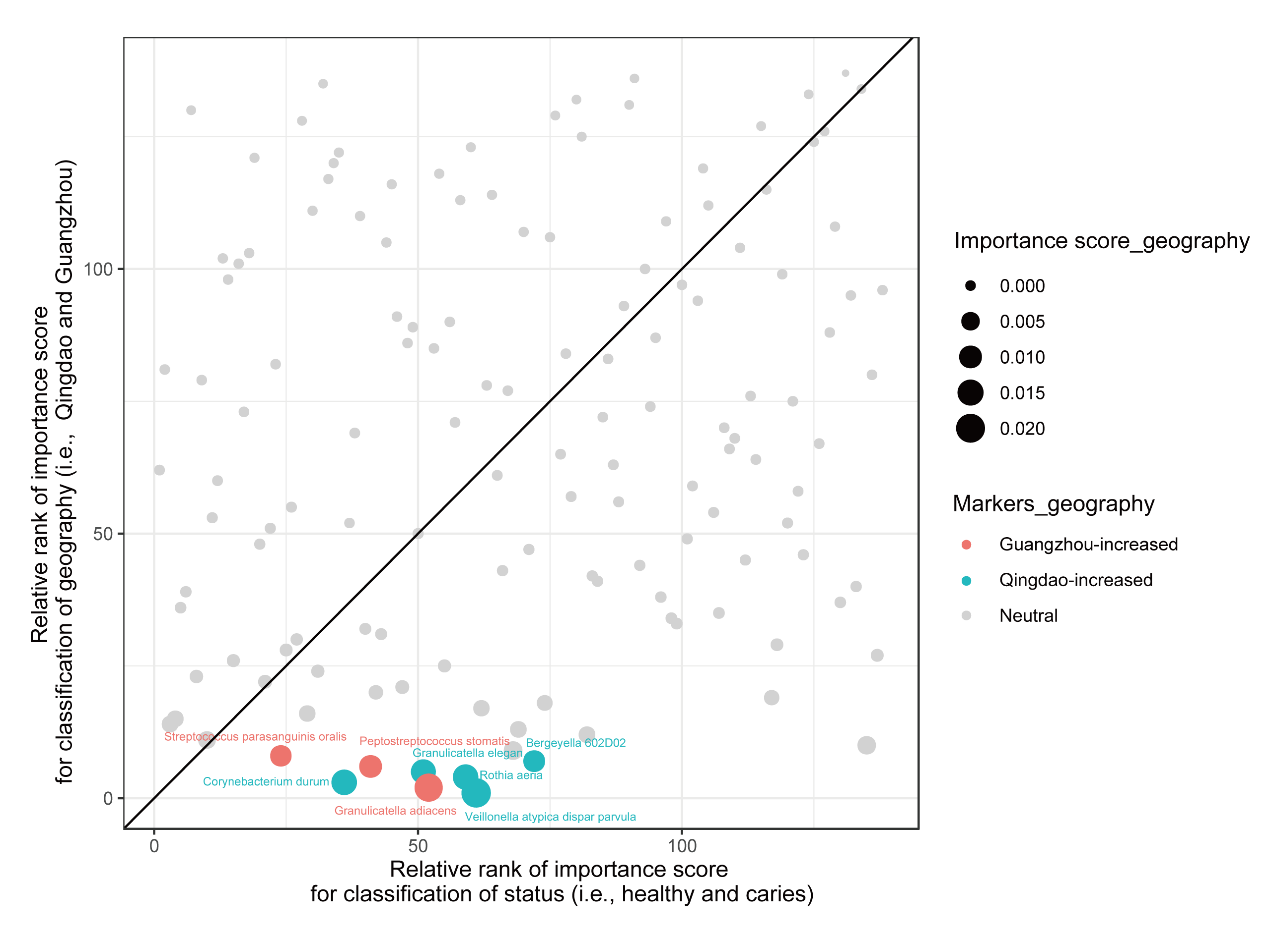


**Supplementary Figure 8. Contributions of the eight most discriminant species to microbiota-based classification of host status and geography.** The most discriminatory taxa (N=8) of geography do not show correlated with caries state. The scatterplot shows the relative rank of microbial markers in both Random Forest models for classifying disease status and geographic locations. Dots on the reference line which slope=1 suggests a taxon is equally important to both models.


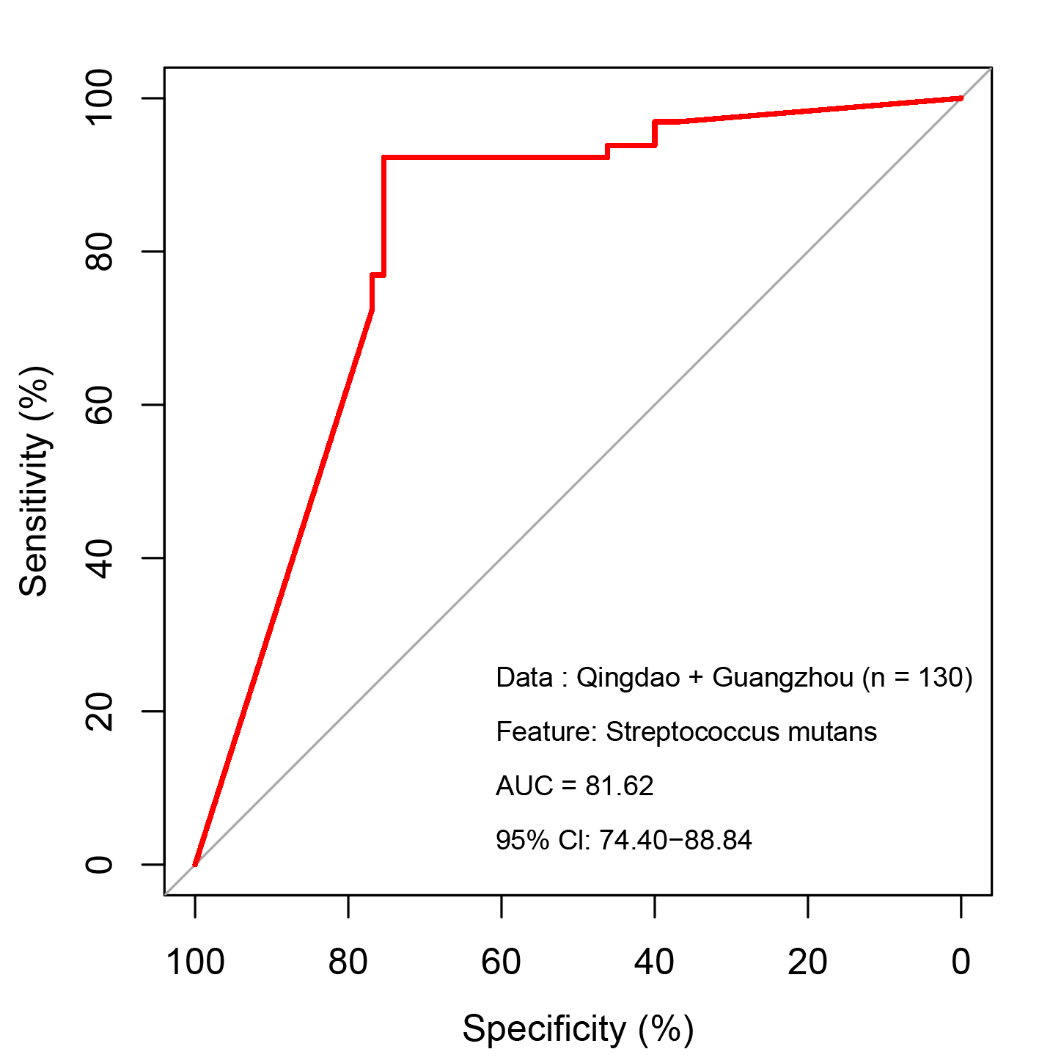


**Supplementary Figure 9. Caries diagnosis model based on the one species of *Streptococcus mutans* after ruling out eight geography-specific signatures.** Performance of status prediction using only *Streptococcus mutans* as a predictor, the simplified Random Forest model led to lower, yet still decent and meaning performance model (AUC=81.62%).


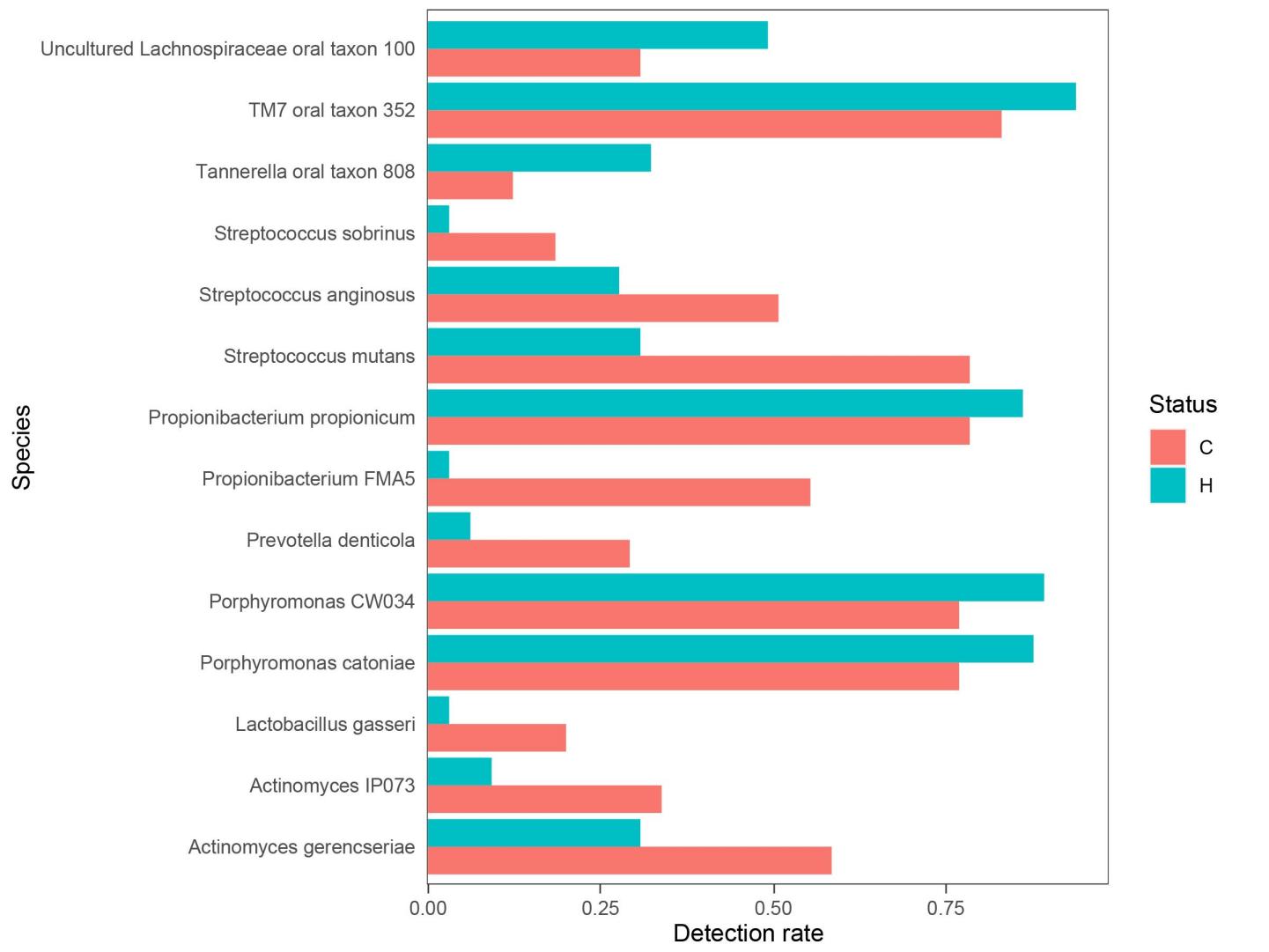


**Supplementary Figure 10. The frequency of fourteen species occurrence in healthy sample and caries samples.** None of 14 species could be detected in all the samples. *Streptococcus mutans, Actinomyces gerencseriae, Propionibacterium FMA5, Streptococcus anginosus, Actinomyces IP073, Prevotella denticola, Lactobacillus gasseri, Streptococcus sobrinus* were detected with a high rate in C group while species of *Tannerella oral taxon 808, Uncultured Lachnospiraceae oral taxon 100, Propionibacterium propionicum, Porphyromonas catoniae, Porphyromonas CW034, TM7 oral taxon 352* were detected with a high rate in H group.

**Table S1. Characteristics of the recruited participants (Qingdao)**

| Sample ID | dmft index | DMFT index | dmft + DMFT  index | Status |
| --- | --- | --- | --- | --- |
| MC1 | 7 | 1 | 8 | C |
| MC10 | 8 | 2 | 10 | C |
| MC12 | 10 | 1 | 11 | C |
| MC13 | 10 | 1 | 11 | C |
| MC14 | 9 | 0 | 9 | C |
| MC15 | 10 | 1 | 11 | C |
| MC16 | 9 | 1 | 10 | C |
| MC17 | 10 | 2 | 12 | C |
| MC18 | 10 | 3 | 13 | C |
| MC19 | 8 | 2 | 10 | C |
| MC2 | 10 | 2 | 12 | C |
| MC20 | 9 | 0 | 9 | C |
| MC21 | 7 | 0 | 7 | C |
| MC23 | 8 | 1 | 9 | C |
| MC27 | 9 | 1 | 10 | C |
| MC28 | 9 | 1 | 10 | C |
| MC29 | 10 | 2 | 12 | C |
| MC3 | 8 | 0 | 8 | C |
| MC30 | 9 | 2 | 11 | C |
| MC31 | 10 | 3 | 13 | C |
| MC32 | 10 | 1 | 11 | C |
| MC33 | 9 | 0 | 1 | C |
| MC35 | 10 | 3 | 13 | C |
| MC36 | 9 | 1 | 10 | C |
| MC39 | 8 | 0 | 8 | C |
| MC4 | 9 | 0 | 9 | C |
| MC41 | 8 | 1 | 9 | C |
| MC42 | 9 | 1 | 10 | C |
| MC43 | 7 | 0 | 7 | C |
| MC44 | 7 | 1 | 8 | C |
| MC45 | 8 | 0 | 8 | C |
| MC46 | 10 | 2 | 12 | C |
| MC47 | 9 | 1 | 10 | C |
| MC48 | 8 | 0 | 8 | C |
| MC49 | 8 | 1 | 9 | C |
| MC5 | 6 | 1 | 7 | C |
| MC50 | 9 | 0 | 9 | C |
| MC51 | 10 | 3 | 13 | C |
| MC52 | 9 | 2 | 11 | C |
| MC53 | 8 | 0 | 8 | C |
| MC54 | 8 | 1 | 9 | C |
| MC55 | 9 | 2 | 11 | C |
| MC56 | 10 | 2 | 12 | C |
| MC57 | 11 | 1 | 12 | C |
| MC58 | 7 | 0 | 7 | C |
| MC59 | 10 | 1 | 11 | C |
| MC6 | 8 | 0 | 8 | C |
| MC60 | 9 | 0 | 9 | C |
| MH1 | 0 | 0 | 0 | H |
| MH10 | 0 | 0 | 0 | H |
| MH11 | 0 | 0 | 0 | H |
| MH12 | 0 | 0 | 0 | H |
| MH13 | 0 | 0 | 0 | H |
| MH14 | 0 | 0 | 0 | H |
| MH15 | 0 | 0 | 0 | H |
| MH16 | 0 | 0 | 0 | H |
| MH17 | 0 | 0 | 0 | H |
| MH18 | 0 | 0 | 0 | H |
| MH19 | 0 | 0 | 0 | H |
| MH2 | 0 | 0 | 0 | H |
| MH22 | 0 | 0 | 0 | H |
| MH23 | 0 | 0 | 0 | H |
| MH24 | 0 | 0 | 0 | H |
| MH25 | 0 | 0 | 0 | H |
| MH26 | 0 | 0 | 0 | H |
| MH27 | 0 | 0 | 0 | H |
| MH28 | 0 | 0 | 0 | H |
| MH29 | 0 | 0 | 0 | H |
| MH3 | 0 | 0 | 0 | H |
| MH30 | 0 | 0 | 0 | H |
| MH31 | 0 | 0 | 0 | H |
| MH32 | 0 | 0 | 0 | H |
| MH33 | 0 | 0 | 0 | H |
| MH34 | 0 | 0 | 0 | H |
| MH35 | 0 | 0 | 0 | H |
| MH36 | 0 | 0 | 0 | H |
| MH37 | 0 | 0 | 0 | H |
| MH38 | 0 | 0 | 0 | H |
| MH39 | 0 | 0 | 0 | H |
| MH4 | 0 | 0 | 0 | H |
| MH40 | 0 | 0 | 0 | H |
| MH41 | 0 | 0 | 0 | H |
| MH42 | 0 | 0 | 0 | H |
| MH43 | 0 | 0 | 0 | H |
| MH44 | 0 | 0 | 0 | H |
| MH46 | 0 | 0 | 0 | H |
| MH47 | 0 | 0 | 0 | H |
| MH48 | 0 | 0 | 0 | H |
| MH49 | 0 | 0 | 0 | H |
| MH5 | 0 | 0 | 0 | H |
| MH50 | 0 | 0 | 0 | H |
| MH51 | 0 | 0 | 0 | H |
| MH52 | 0 | 0 | 0 | H |
| MH54 | 0 | 0 | 0 | H |
| MH55 | 0 | 0 | 0 | H |
| MH56 | 0 | 0 | 0 | H |
| Mean | 8.81±1.10 | 1.06±0.93 | 9.70±2.17 |  |

(C, Caries group; H, Healthy group.)

**Table S2. Characteristics of the recruited participants (Guangzhou)**

| Sample ID | dmft index | DMFT index | dmft + DMFT index | Status |
| --- | --- | --- | --- | --- |
| MC101 | 6 | 0 | 6 | C |
| MC102 | 8 | 1 | 9 | C |
| MC104 | 7 | 1 | 8 | C |
| MC105 | 6 | 0 | 6 | C |
| MC85 | 9 | 1 | 10 | C |
| MC86 | 10 | 2 | 12 | C |
| MC87 | 8 | 0 | 8 | C |
| MC88 | 8 | 1 | 9 | C |
| MC89 | 9 | 1 | 10 | C |
| MC91 | 8 | 0 | 8 | C |
| MC92 | 8 | 1 | 9 | C |
| MC94 | 9 | 2 | 11 | C |
| MC95 | 9 | 1 | 10 | C |
| MC96 | 8 | 1 | 9 | C |
| MC97 | 10 | 1 | 11 | C |
| MC98 | 7 | 0 | 7 | C |
| MC99 | 6 | 1 | 7 | C |
| MH58 | 0 | 0 | 0 | H |
| MH60 | 0 | 0 | 0 | H |
| MH61 | 0 | 0 | 0 | H |
| MH64 | 0 | 0 | 0 | H |
| MH65 | 0 | 0 | 0 | H |
| MH66 | 0 | 0 | 0 | H |
| MH67 | 0 | 0 | 0 | H |
| MH68 | 0 | 0 | 0 | H |
| MH69 | 0 | 0 | 0 | H |
| MH70 | 0 | 0 | 0 | H |
| MH80 | 0 | 0 | 0 | H |
| MH81 | 0 | 0 | 0 | H |
| MH82 | 0 | 0 | 0 | H |
| MH83 | 0 | 0 | 0 | H |
| MH84 | 0 | 0 | 0 | H |
| MH85 | 0 | 0 | 0 | H |
| MH86 | 0 | 0 | 0 | H |
| Mean | 8.0±1.27 | 0.82±0.63 | 8.82±1.74 |  |

(C, Caries group; H, Healthy group.)
